# Current and Emerging Parenteral and Peroral Medications for Weight Loss: A Narrative Review

**DOI:** 10.3390/diseases13050129

**Published:** 2025-04-22

**Authors:** Abdullah Al Lawati, Ayman Alhabsi, Rhieya Rahul, Maria-Luisa Savino, Hamed Alwahaibi, Srijit Das, Hanan Al Lawati

**Affiliations:** 1 Sultan Qaboos University Hospital, Al-Khoud 123, P.O. Box 50, Muscat 123, Oman; abdlawati1@gmail.com; 2 Department of Medicine, Royal College of Surgeons, 123 St Stephen’s Green, D02 YN77 Dublin, Ireland; aymanalhabsi@gmail.com (A.A.); rhieyarahul@gmail.com (R.R.); marialuisasavin23@rcsi.com (M.-L.S.); hamed253@icloud.com (H.A.); 3 Department of Human and Clinical Anatomy, College of Medicine and Health Sciences, Sultan Qaboos University, Al-Khoudh 123, P.O. Box 50, Muscat 123, Oman; s.das@squ.edu.om; 4 Pharmacy Program, Department of Pharmaceutics, Oman College of Health Sciences, P.O. Box 393, Muscat 113, Oman

**Keywords:** obesity, weight loss, parenteral, peroral, medications, emerging, pharmacological, surgical

## Abstract

Obesity is a growing global health challenge, necessitating effective treatment options beyond lifestyle interventions. This narrative review explores established and emerging pharmacotherapies for weight management, including parenteral agents like Liraglutide, Semaglutide, Setmelanotide, and Tirzepatide, as well as peroral medications such as Phentermine, Phentermine/Topiramate, Bupropion/Naltrexone, Orlistat, and Metformin. Newer treatments like Cagrilintide and Bimagrumab show promise for enhancing weight loss outcomes. Parenteral GLP-1 receptor agonists demonstrate superior efficacy compared to traditional peroral medications, with gastrointestinal side effects being the most common. Artificial intelligence presents intriguing opportunities to enhance weight loss strategies; however, its integration into clinical practice remains investigational and requires rigorous clinical validation. While current anti-obesity medications deliver significant benefits, future research must determine the efficacy, safety, and cost-effectiveness of AI-driven approaches. This includes exploring how AI can complement combination therapies and tailor personalized interventions, thereby grounding its potential benefits in robust clinical evidence. Future directions will focus on integrating AI into clinical trials to refine and personalize obesity management strategies.

## 1. Introduction

Obesity is a complex multifactorial disease that results in the collection of excess fat in the body, impairing overall health [1]. According to the World Health Organization (WHO), obesity is defined as a body mass index (BMI) ≥ 30 [2]. Obesity has been a growing concern over the past 50 years. In 1975, 100 million adults were living with obesity [3]. This number nearly quadrupled to 396 million by 2005, reached 609 million by 2015, and rose to 800 million in 2020, which increased to 890 million in 2022 [4,5,6,7]. The escalating burden of obesity is reflected in the disability-adjusted life years (DALYs) and mortality attributed to obesity. DALYs is a measurement of the overall disease burden and calculated by adding the number of years of life lost because of the disease to the years lived with disability [8,9]. Obesity-associated DALY rates were 160.3 million worldwide in 2019, making obesity responsible for 5.89% of global DALYs. In the same year, obesity was directly responsible for 8.52% of overall mortality, resulting in 5.03 million deaths. Compared to those in 1990, obesity-related DALYs and mortality nearly doubled, which highlights the increasing impact of obesity on global health [8,9].

Moreover, obesity is associated with multiple chronic diseases, including cardiovascular diseases (CVDs), type 2 diabetes (T2D), hypertension and dyslipidemia, with CVDs and T2D being the most strongly linked to obesity [10,11]. Interestingly, many of these comorbidities follow a dose-response relationship with obesity, meaning that the likelihood of being affected by them increases with higher body mass index (BMI) [12]. For example, one study shows that the likelihood of developing diabetes increased 17 times in individuals with a BMI ≥ 35 compared to those with a BMI between 25 and 29 [12]. In addition, a 1-unit gain in BMI can result in a 4% increase in the risk of atrial fibrillation [13], and a 5-unit increase in BMI can increase the risk of coronary heart disease by 29% [14]. Also, an additional 10 kg body weight is linked to a 3.0 mmHg increase in systolic blood pressure (SBP) and a 2.3 mmHg increase in diastolic blood pressure (DBP) [15].

Given the rising prevalence and health impact of obesity, effective management strategies are crucial. Current clinical guidelines for obesity management are based on the joint recommendations of the American Heart Association (AHA), American College of Cardiology (ACC), and The Obesity Society (TOS) from 2013, as well as the 2016 guidelines by the American Association of Clinical Endocrinologists (AACE) and American College of Endocrinology (ACE). AHA/ACC/TOS recommends a 5–10% reduction in the baseline weight in 6 months as an initial aim, which is enough to produce clinical benefits. A 5% weight reduction can considerably reduce the blood concentrations of substances associated with cardiometabolic diseases and enhance beta-cell function and insulin sensitivity of multiple organs, including the liver and adipose tissue [16,17]. Moreover, a 5–10% weight reduction has been shown to reduce SBP and DBP by 5 mmHg [18] and enhance metabolic diseases [16]. Lifestyle interventions are recommended for patients with BMI between 25 and 29.9 without obesity-related complications. The recommended daily calorie intake is 1200–1500 for females and 1500–1800 for males. Additionally, it is advised to engage in aerobic activity for 150–300 min at moderate intensity or 75–150 min at vigorous intensity per week, along with at least two strength exercise sessions. Pharmacological interventions are introduced to patients with a BMI ≥ 30 or a BMI ≥ 27 if they have obesity-associated comorbidity or if lifestyle interventions fail. Combining pharmacological and lifestyle interventions can lead to more significant weight reduction than lifestyle changes alone. Bariatric surgery is indicated for patients with a BMI ≥ 40 or a BMI ≥ 35 with obesity-associated complications [19,20,21].

**Figure 1:**
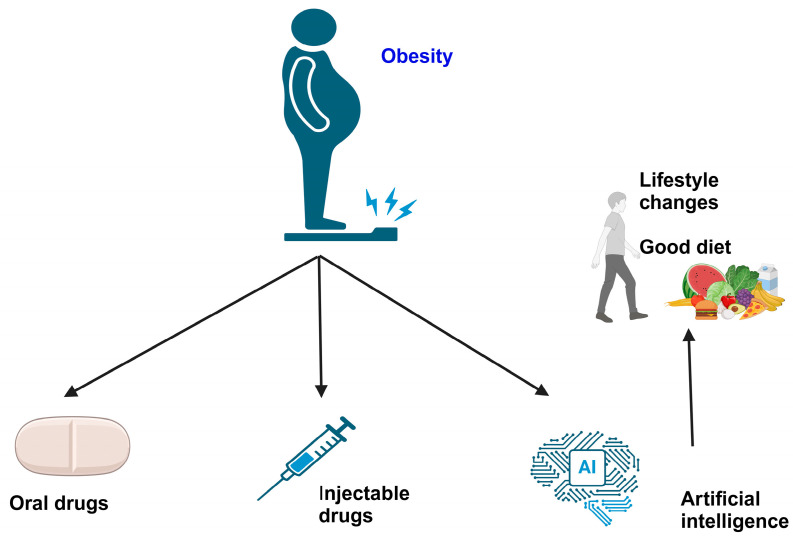
Schematic diagram showing the approach to treating obesity. Created with BioRender software.

Several reviews have examined current and emerging pharmacological approaches to weight loss; however, gaps remain in addressing other modalities such as lifestyle modifications and surgical procedures, as well as in directly comparing these strategies. Additionally, there is a need for more discussion on the role of artificial intelligence (AI) in obesity treatment, along with safety considerations and regulatory recommendations. This narrative review will address these gaps while exploring some emerging anti-obesity medications and both current and novel parenteral and peroral medications for weight loss with emphasis on their mechanisms of action, efficacy, and safety. The parenteral drugs in this review—such as Liraglutide, Semaglutide, Setmelanotide, and Tirzepatide—have gained attention due to their ability to modulate appetite and result and significant weight loss. The approach to treating obesity is also summarized in Figure 1.

## 2. Parenteral Medications for Weight Loss

Both surgical and pharmacological strategies have been developed to optimize therapeutic outcomes in managing chronic obesity. Among pharmacological treatments, parenteral drugs such as Liraglutide, Semaglutide, Setmelanotide, and Tirzepatide have promising potential.

### 2.1. Liraglutide (Saxenda)

Liraglutide, a glucagon-like peptide-1 (GLP1) receptor agonist, was initially developed for diabetes management. During clinical trials, it demonstrated potential in promoting weight reduction, leading to its approval for chronic weight management at a subcutaneous administration dose of 3 mg once daily. It is intended for long-term use, and studies have assessed its safety and efficacy over treatment periods of 1 to 2 years. Structurally, Liraglutide (Saxenda^®^) shares a 97% structural similarity with native human GLP-1 [22,23]. It includes a substitution of arginine for lysine at position 34 and a fatty acid side chain attached to lysine at position 26. It has a prolonged half-life of 10–14 h due to its additional albumin-binding fatty-acid side chain, which reduces its degradation by dipeptidyl peptidase-4 (DPP-4) and prolongs its half-life compared to endogenous GLP1 [24,25,26,27]. Over the treatment period of two years, Liraglutide was shown to reduce body weight by an average of 4.7%, primarily by activating GLP-1 receptors in the gastrointestinal and nervous systems. This interaction reduced energy intake, delayed gastric emptying, and increased satiety, contributing to weight loss [26,28]. Liraglutide crosses the blood-brain barrier through transcytosis, facilitated by tanycytes—specialized ependymoglial cells—and acts on hypothalamic GLP-1 receptors [29]. Once it is bound to the GLP-1 receptor, proopiomelanocortin and cocaine- and amphetamine-regulated transcript (POMC/CART) neurons are stimulated, and both neuropeptide Y (NPY) and agouti-related peptide (AgRP) are inhibited. This central mechanism helps regulate feelings of hunger and satiety, food intake, body weight, fat mass, and energy metabolism, including fatty acid oxidation [30]. Furthermore, Liraglutide has effectively reduced central adiposity, as indicated by decreased waist circumference and waist-to-hip ratio. It also significantly reduces android fat distribution.

Beyond weight loss, Liraglutide has demonstrated cardiovascular benefits, including lowering triglycerides (TRG) levels and improving the TRG/HDL ratio. In terms of the safety profile of Liraglutide, studies have reported a low risk of hypoglycemia in chronically obese, non-diabetic patients treated with 3 mg of Liraglutide, with an incidence of 1.3% compared to 1.0% in the placebo group [31]. Moreover, Liraglutide has shown promising therapeutic effects in obesity-related conditions, including obstructive sleep apnea. A clinical study demonstrated that 3 mg of Liraglutide significantly reduced the apnea–hypopnea index by 12.2 per hour, compared to 6.1 per hour in the placebo group. This improvement was due to the more significant weight reduction observed in the Liraglutide group (5.7%), compared to (1.6%) in the placebo group [32]. However, typical side effects of Liraglutide treatment include nausea (48.4%), vomiting (23.2%), and diarrhea (23.1%) [26,31,33,34,35,36]. Compared to other GLP-1 receptor agonists, Liraglutide has a shorter half-life, potentially leading to more frequent dosing and the return of hunger sensation between doses. In contrast, Semaglutide requires less frequent dosing with its longer half-life [37]. Notably, Liraglutide increases the risk of pancreatitis, with a reported incidence of 4%. Although this risk is relatively low, it is higher than other GLP-1 receptor agonists. As such, Liraglutide should be used with caution in patients at higher risk for pancreatitis (e.g., alcohol abuse, history of pancreatitis, cholelithiasis) [38,39].

### 2.2. Semaglutide (Wegovy)

Semaglutide, marketed under different brand names, was initially developed for treating type 2 diabetes (Ozempic^®^). However, recent studies have demonstrated its effectiveness in managing obesity, leading to the development of Wegovy^®^ for this purpose. Clinical trials have shown promising therapeutic effects of Wegovy^®^ in managing obesity, with evidence indicating that it can lead to a 15% reduction in body weight over 68 weeks [40,41]. Consequently, the U.S. Food and Drug Administration (FDA) has approved Semaglutide for chronic obesity at a dose of 2.4 mg once weekly. Semaglutide is a synthetic analogue of glucagon-like peptide 1 (GLP-1), sharing 94% amino acid homology with native GLP 1 [42]. GLP-1 is naturally synthesized by L-cells found in the small intestine and colon. Upon binding to GLP-1 receptors in tissues such as the brain, pancreas, and gut, it stimulates insulin secretion, inhibits glucagon release, slows gastric emptying, and enhances feelings of satiety, reducing food intake [43,44,45]. These combined actions are key to Semaglutide’s efficacy in weight reduction, as native GLP-1 has a short half-life of 2–11 min due to rapid degradation by the enzyme dipeptidyl peptidase-4 (DPP-4) [46]. To overcome the challenge of rapid degradation, Semaglutide was designed with a longer half-life of approximately one week, making it resistant to DPP-4 degradation and allowing for sustained therapeutic effects [42].

This extended half-life is achieved through two key modifications: the addition of a fatty diacid chain at position 26, which increases binding affinity to albumin, and the substitution of alanine with α-aminoisobutyric acid at position 8, making the molecule more resistant to DPP-4 degradation [41]. Additionally, evaluating the safety profile and side effects of Semaglutide is crucial for its clinical therapeutic use. Clinical trials have reported that Semaglutide is associated with gastrointestinal side effects such as nausea, vomiting, and diarrhea. In phase 3 trials, subcutaneous Semaglutide over 30 weeks induced nausea in 11.4 to 20% of patients (compared to 3.3–8% in the placebo group), vomiting in 4 to 11.5% (placebo 2–3%), and diarrhea in 4.5 to 11.3% (placebo 1.5–6%) [47,48,49]. Notably, these gastrointestinal side effects were reduced by 23% when dose escalation was carried out over 8 weeks instead of 4 weeks [50,51]. In terms of serious adverse events, a study observed acute gallbladder disease happening in approximately 4% of participants receiving once-weekly subcutaneous Semaglutide (2.4 mg). However, no cases of acute renal failure, diabetic retinopathy, or severe hypoglycemia were reported. Semaglutide has also been shown to significantly improve cardiometabolic risk factors, including reduced waist circumference and levels of glycated hemoglobin and lipids [52]. Notably, a meta-analysis of 37 studies found no significant association between Semaglutide and pancreatic or thyroid cancer. The risk of all neoplasms, including benign, malignant, and unspecified tumors, was similar between the Semaglutide and placebo groups, with no statistically significant differences observed [53].

### 2.3. Setmelanotide (Imcivree)

Setmelanotide (IMCIVREE^®^) is a melanocortin-4 (MC4) receptor agonist that was approved by the FDA in 2020 for the treatment of chronic genetic obesity [54,55]. It is designed for long-term use, and clinical trials have evaluated its safety and efficacy over treatment periods of one year or longer. The subcutaneous parenteral form of Setmelanotide is specifically indicated in patients with genetic defects in the MC4R activation pathway, such as variants in the proopiomelanocortin (POMC), proprotein convertase subtilisin/kexin type 1 (PCSK1), or leptin receptor (LEPR) genes. These defects impair the body’s natural weight regulation mechanisms, typically confirmed through genetic testing [54,55,56,57]. The MC4R activation pathway begins with leptin binding to LEPR, activating neurons that express POMC. POMC is cleaved by PCSK1, resulting in the production of ⍺-melanocyte-stimulating hormone (α-MSH), the endogenous ligand for the MC4 receptor. Activation of MC4 receptors plays a key role in regulating body weight [58]. Therefore, genetic deficiencies in any part of this pathway can lead to chronic obesity, which Setmelanotide can treat. Structurally, Setmelanotide is a cyclic peptide containing eight amino acids with a disulfide bond linking two cysteines near the termini. The molecule also features phenylalanine in the D-configuration within the conserved HFRW motif [59]. Setmelanotide’s effectiveness lies in its biased agonism, which strongly activates PLC-β (phospholipase C-β) via the G-protein Gαq. This unique mechanism allows Setmelanotide to resist antagonism by agouti-related peptide (AgRP), a natural antagonist of MC4R. As a result, Setmelanotide activates MC4R more effectively than the natural ligand α-MSH, leading to greater clinical benefits such as significant weight reduction [60]. Additionally, Setmelanotide has shown therapeutic efficacy in cases of chronic obesity caused by ciliopathic genetic syndromes, such as Bardet–Biedl syndrome (BBS), caused by a mutation in (BBS1-BBS22). In these cases, Setmelanotide bypasses the defective leptin pathway and directly activates the MC4 receptor, ultimately reducing weight [58].

As a result of the activation of the MC4 receptor by Setmelanotide, a variety of positive clinical therapeutic effects can be achieved, including reducing feelings of hunger, lowering calorie intake, and increasing energy expenditure, potentially leading to weight loss [61]. Clinical trials support Setmelanotide’s impact on weight loss. In trials involving patients with LEPR and POMC deficiencies (11 and 10 participants, respectively), 80% of participants in the POMC trial and 45% in the LEPR trial achieved 10% weight loss after one year. Changes in hunger feelings were reported as 27.1% (*p* = 0.0005) in the POMC trial and 43.7% (*p* < 0.0001) in the LEPR trial. Common side effects in the POMC trial included injection site reaction and hyperpigmentation in all participants, nausea in five participants, and vomiting in three. In the LEPR trial, eleven participants experienced injection site reactions, five had skin disorders, and four reported nausea [54]. A separate clinical trial in obese BBS patients showed that 32.3% achieved weight loss after one year of Setmelanotide treatment. The most common side effect reported by participants was skin hyperpigmentation, occurring in 60.5% of participants [62]. Overall, Semaglutide, Liraglutide, and Setmelanotide represent promising parenteral pharmacological agents that have demonstrated significant clinical efficacy in managing obesity. Ongoing studies and clinical trials continue to evaluate their long-term safety, effectiveness, and potential for broader applications in obesity treatment.

### 2.4. Tirzepatide

Tirzepatide is the most recent FDA-approved drug for treating type 2 diabetes mellitus (T2DM) and obesity. It is intended for long-term use, with studies assessing its effects over approximately 72 weeks [63,64]. Tirzepatide is a GLP-1 and GIP receptor agonist that binds and activates these receptors, causing decreased appetite and lowering food intake [64]. It was approved by the FDA in November 2023 for use in chronic weight management, specifically in combination with dietary modifications (a reduced calorie diet) and increased physical activity [63]. Tirzepatide’s dual mechanism of action makes it a promising new weight-loss medication option. In a double-blind, randomized, multicenter, placebo-controlled phase 3 trial, patients who received once-weekly Tirzepatide showed significant reductions in body weight [64]. Specifically, patients receiving 10 mg and 15 mg doses of Tirzepatide achieved an average weight loss of 12.8% and 14.7%, respectively, over 72 weeks, demonstrating a therapeutic effect on body weight [64]. In another phase 3 trial, patients with type 2 diabetes were either assigned Tirzepatide or Semaglutide, and those who received Tirzepatide had a superior mean change in glycated hemoglobin [65]. In this trial, patients who received Tirzepatide at doses of either 5 mg, 10 mg, or 15 mg had a mean change in glycated hemoglobin by –2.01, –2.24, or –2.30, respectively, compared to Semaglutide which resulted in a mean difference of –1.86%. Tirzepatide has been studied in patients with only obesity, with obesity and type 2 diabetes, and patients with only diabetes, and in all, it has shown strengths as a practical option for weight loss. An exciting part of Tirzepatide research has been the improvement in insulin responses in patients with type 2 diabetes over Semaglutide [66]. Tirzepatide was shown to improve bodily insulin secretion in patients who had previously been unresponsive to similar medications while causing more considerable weight loss than Semaglutide [66]. In addition to the weight loss findings of these studies, Tirzepatide has shown little increase in adverse events compared to similar medications [67]. Based on the findings of these studies, Tirzepatide is a compelling new drug due to its action on patients’ insulin levels and weight loss results, offering optimism for the future of weight loss medications.

**Figure 2:**
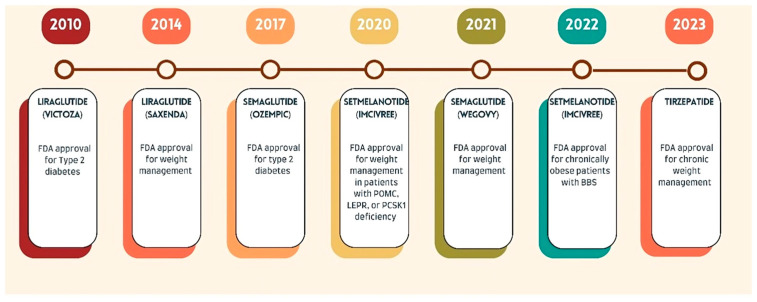
Timeline of FDA approvals for weight loss parenteral medications.

Figure 2 demonstrates the timeline of FDA approvals for weight loss parenteral medications [68,69,70,71,72].

## 3. Peroral Medications for Weight Loss

In addition to parenteral medications, various peroral medications are also available for weight loss.

### 3.1. Phentermine

Phentermine is a primary amine, Schedule 4 drug (low risk of abuse or dependence) that was shown in the 1970s to inhibit the metabolism of serotonin by monoamine oxidase but was never labelled as a monoamine oxidase (MAO) inhibitor [73,74]. Phentermine was FDA-approved in 1959, and it became widely used in 1960. It was initially combined with fenfluramine and dexfenfluramine; however, this was discontinued after finding abnormal values amongst 30% of consumers. Later, it was approved in 2012 to be used alone or in combination with topiramate [75].

Pharmacological interventions such as the sympathomimetic amine phentermine (used in some countries longer term under specialist supervision) remain one of the most prescribed short-term treatments for obesity. The drug primarily increases norepinephrine levels within the hypothalamus, leading to decreased appetite and increased satiety signals [76]. Phentermine shows notable effectiveness, with clinical studies demonstrating that 60% of patients lose at least 5% of their total body weight within three months of treatment [77,78,79,80,81]. The medication shows particular promise in the early weeks of treatment, with patients experiencing an average weight loss of 3.6–7.2 kg over 12 weeks [77,78,79,80,81], making it a go-to short-term solution for obesity management. Its regulatory classification as a Schedule 4 drug indicates its controlled but recognized medical use [77,78,79,80,81].

Although its effectiveness has been established, regulatory restrictions respond to safety concerns. Insomnia, dry mouth, and constipation have been reported in 15–30% of subjects as the most frequent adverse effects [79]. However, the cardiovascular effects of increased heart rate and blood pressure are more concerning, prompting a thoughtful selection of patients. These risks on the cardiovascular level have made those patients with uncontrolled hypertension, a known history of hyperthyroidism, or previously diagnosed cardiac disease contraindicated [80].

The similarities between phentermine and amphetamines are such that the Drug Enforcement Administration of America classifies it as a Schedule 4 controlled substance, meaning its prescription use is regulated. There are guidelines concerning treatment that suggest not treating patients for longer than 12 weeks, although some studies demonstrate safety over the long term when used under close medical supervision [81]. For example, patient selection criteria include extensive cardiovascular screening and continuous monitoring of vital signs during treatment. In conclusion, phentermine remains a widely prescribed short-term pharmacological treatment for obesity due to its ability to suppress appetite and induce modest weight loss, particularly in the early weeks of treatment.

### 3.2. Phentermine/Topiramate (Qsymia)

Topiramate is a hexose derivative 2,3:4,5-di-O-isopropylidene-β-D-fructopyranose, in which the hydroxy group has been converted to the corresponding sulfamate ester. It blocks voltage-dependent sodium channels and is used in treating migraines. Additionally, it has a role as an anticonvulsant and a sodium channel blocker. It was initially approved by the FDA in 1996 and was approved in 2004 for migraine prevention. It was discovered by chance when attempting to formulate an anti-diabetic drug. In 2012, it was approved for combination with phentermine (refer to the previous subtopic for more information) for chronic weight management therapy in adults [82]. Phentermine–topiramate is a Schedule 4 drug [83]. The extended-release fixed-dose combination of phentermine and topiramate represents a significant advancement in obesity pharmacotherapy. This combination combines two pharmacologically complementary mechanisms of action: phentermine, a sympathomimetic amine anorectic, as mentioned in the previous paragraph, works by increasing norepinephrine levels within the hypothalamus, leading to a decrease in appetite; and topiramate, which enhances satiety and energy expenditure [76,84]. This synergistic effect permits the administration of lower dosages needed from each component to avoid adverse effects at decreased therapeutic efficacy.

Clinical trials demonstrate more significant weight loss with the combination than monotherapy with either drug. In the 56-week CONQUER trial of overweight and obese adults (*n* = 2487), participants receiving the highest approved dose (15/92 mg) experienced mean weight reductions of 9.8% after 56 weeks compared to 1.2% with the placebo [85]. In some subgroup analyses, as much as 70% of patients taking high-dose PHEN/TPM ER achieved ≥5% weight loss, with 48% achieving loss ≥10% from the baseline [86]. The SEQUEL study further established its long-term efficacy, showing sustained weight loss over 2 years and improvements in cardiovascular risk factors [87]. This combination therapy is also associated with beneficial outcomes for obesity-related comorbidities, including notable reductions in sleep apnea severity and significant enhancements in quality-of-life parameters.

Side effects profile revealed dose-dependent adverse events, with the most commonly reported being paresthesia, dry mouth, constipation, and insomnia. Other side effects include cognitive function, affecting attention and memory impairment, necessitating gradual dose titration and regular monitoring [81]. Given its teratogenicity, strict pregnancy testing and contraception requirements must apply to women of childbearing potential. Overall, phentermine–topiramate represents a significant advancement in obesity management, offering enhanced weight loss and metabolic benefits. However, it requires careful patient monitoring to manage its side effects and ensure safe use, particularly in women of reproductive age.

### 3.3. Bupropion/Naltrexone (Contrave)

Bupropion is an aminoketone propiophenone carrying a tert-butylamino group at position two and a chloro substituent at position three on the phenyl ring. The mechanism of action of bupropion is as a dopamine uptake inhibitor and norepinephrine uptake inhibitor. The physiological effect of bupropion is increased dopamine activity and norepinephrine activity. It is a secondary amino compound, a member of monochlorobenzenes, and an aromatic ketone [88]. The medication was first patented by Burroughs Welcome in 1974 and approved by the FDA in 1985 [89]. Naltrexone is an organic heteropentacyclic compound that is naloxone substituted in which a cyclopropyl methyl group replaces the allyl group attached to the nitrogen. A mu-opioid receptor antagonist, it is used to treat alcohol dependence. It is an organic heteropentacyclic compound, a morphine-like compound, and a member of cyclopropanes. It is a derivative of noroxymorphone that is the N-cyclopropyl methyl congener of naloxone [90]. It was developed in 1963 for alcohol use disorder and opioid dependence, patented in 1967, and received FDA approval in 1984 [91].

The combination of bupropion, a norepinephrine–dopamine reuptake inhibitor, and naltrexone, an opioid receptor antagonist, provides a novel approach to weight management through dual modulation of appetite and reward pathways. Bupropion activates proopiomelanocortin (POMC) neurons of the hypothalamus, suppressing feeding, increasing expenditure and improving glucose tolerance. At the same time, naltrexone blocks β-endorphin-dependent POMC auto-inhibition and thus maintains prolonged activity for anorexigenic pathways, suppressing appetite [92]. At 56 weeks, patients on the prescribed dose in the phase III COR-I study lost an average of 6.1% of their body weight, while those on the placebo lost 1.3% [93]. A post hoc study revealed that the greatest effects occurred within 28 weeks after the start of treatment, with approximately 48% of treated patients seeing weight loss greater than 5% [94]. Patient tolerability varies; 29.8% experienced nausea, while secondary adverse reactions included headache (17.6%) and constipation (19.2%). The high rate of dropouts due to adverse events, approaching 24% in clinical trials, requires cautious patient counselling and slow dose escalation [95]. The combination has selectively found use in patients with mood disorders (in addition to substance abuse or eating disorders) and atypical depression, potentially due to bupropion’s anti-depressant properties. Long-term cardiovascular outcomes have been evaluated through the LIGHT study. However, it was halted ahead of schedule after it fell short of its primary CVD outcome goal to judge long-term cardiovascular safety (for which preliminary data instead suggested no added cardiovascular danger). Blood pressure control remains important because modest increases have been reported, especially in the first 2 weeks of therapy [96]. In conclusion, the combination of bupropion and naltrexone offers an innovative approach to weight management by modulating both appetite and reward pathways by activating POMC neurons and inhibiting opioid receptors. Clinical trials have demonstrated its efficacy, with significant weight loss observed in patients, though tolerability and adherence may be challenged by adverse effects such as nausea, headache, and constipation.

### 3.4. Orlistat (Alli, Xenical)

Orlistat is a carboxylic ester resulting from the formal condensation of the carboxy group of N-formyl-L-leucine with the hydroxy group of (3S,4S)-3-hexyl-4-[(2S)-2-hydroxytridecyl] oxetane-2-one. It is a beta-lactone, an L-leucine derivative, a member of formamides, and a carboxylic ester. It is a saturated derivative derived from the endogenous lipstatin in Streptomyces toxytricinihas [97]. It has the role of a triacylglycerol inhibitor, bacterial metabolite, fatty acid synthase inhibitor, and anti-obesity agent [98]. The weight loss drug orlistat [Xenical] offers a novel peripheral mechanism in treating obesity as an inhibitor of gastrointestinal lipase. It was initially approved by the US FDA in 1999 as a prescription product [99].

Orlistat has no effects on the central nervous system and reduces dietary fat absorption by up to 30%, which results in a negative energy balance [100,101]. It is now the only FDA-approved weight loss drug for use in teenagers between the ages of 12 and 16. It is available as an over the counter (Alli) and prescription (Xenical) product with strengths of 60 mg and 120 mg, respectively. A 1-year weight loss in the range of 2.9 to 3.4 kg is greater than a placebo after a year of treatment, according to meta-analyses of randomized controlled studies, which show modest but consistent efficacy [101]. The XENDOS study’s four-year randomized data showed that treated individuals had a decreased incidence of new-onset type 2 diabetes and sustained weight loss, with 52.8% losing ≥5% bodyweight in one year [102].

Mechanism of action-related gastrointestinal side effects affect about 15–30% of patients. These include oily spotting, flatus with discharge, and fecal urgency when the fat content in the diet is more than 30% per calorie [103]. As such, patient education regarding dietary modification and fat intake restriction continues to be paramount for both efficacy and tolerability. Long-term safety data is robust, with minimal systemic absorption contributing to a favorable cardiovascular risk profile. Nonetheless, a few cases of significant liver injury have occurred that required monitoring of hepatic function [104]. In conclusion, orlistat provides a unique, non-systemic approach to weight management by inhibiting dietary fat absorption, offering modest but sustained weight loss and a favorable cardiovascular risk profile.

### 3.5. Metformin (Off-Label Use)

Metformin hydrochloride is a leading biguanide antihyperglycemic agent that manages high blood sugar levels in type 2 diabetes and is derived from Galega officinalis, a plant otherwise known as goat’s rue. Guanidine was first created in the late 1870s and, when administered to rabbits in 1918, was shown to lower blood glucose levels. Following this, guanidine derivatives were proposed as possible treatments for diabetes, including the aminoguanidine glargine and the more powerful diguanide synthalin. Soon after, toxicity was noted, and by the 1930s, they had been overshadowed by insulin, which had been identified in 1922 and was beginning to be manufactured more widely [105]. It was officially approved in 1994 for treating type 2 diabetes and has since become the most widely prescribed medication [106].

Although, most importantly, it is used off-label for managing type 2 diabetes, metformin has shown promising results in different patient populations concerning weight loss. Its mechanisms of action include reduced hepatic glucose production, enhanced peripheral insulin sensitivity, and modulation of the gut microbiome [107]. Clinical studies indicate metformin causes a slight weight loss in at-risk populations (those who are insulin-resistant/overweight). Meta-analyses suggest that, in overweight or obese participants, a reduction of 1.1–2.6 kg occurs compared to placebo over 6–12 months [108]. The most prominent example was the Diabetes Prevention Program, which showed sustained weight loss over 2.8 years and a more significant impact in patients with higher baseline BMIs [109]. Specific populations demonstrating enhanced benefit include individuals with polycystic ovary syndrome (PCOS), prediabetes, and antipsychotic-induced weight gain. The weight loss is also relatively modest, with a mean reduction of 2.7 kg over six months in patients observed for PCOS during metformin therapy, lending balance to any improvements achieved herein and/or previously reported regarding menstrual regularity and metabolic parameters [110].

In addition, gastrointestinal side effects, including diarrhea and nausea, the latter occurring in 20–30% of cases, can be mitigated by gradually increasing dose titration or using extended-release formulations. Its favorable safety profile, long track record in the clinical use, and potential metabolic effects render metformin an intriguing possibility for specific patient populations, especially those with insulin resistance or prediabetic states [111].

In conclusion, while primarily used for type 2 diabetes, metformin’s modest but beneficial effects on weight loss and metabolic health in specific populations—such as those with insulin resistance, PCOS, and prediabetes—make it a promising off-label option, especially given its well-established safety profile and potential for managing obesity-related conditions.

## 4. Emerging Medications in Clinical Trials

Weight loss medications are an ever-changing field, with several drugs emerging recently. The rise of Semaglutide (Ozempic) has pushed research in this area much further than before. Several promising medications, including Cagrilintide and Bimagrumab, have not yet been fully approved or put on the market, although both have been studied extensively in clinical trials.

### 4.1. Cagrilintide

Cagrilintide is a long-acting amylin analogue. Amylin is a 37 amino acid peptide and an essential pancreatic hormone for maintaining homeostasis [112,113]. The pancreas releases amylin and insulin, which then travel to the brain and induce a feeling of satiety [114]. Cagrilintide, primarily being evaluated for its role in weight loss treatments, has shown efficacy when combined with other medications, such as Semaglutide [115]. In clinical studies, Cagrilintide was distributed via subcutaneous injection, ensuring consistent therapeutic levels throughout the treatment period. In a systematic review of 76 trials involving weight loss management drugs, combining Cagrilintide and Semaglutide led to the highest reduction in weight, with a mean difference of –14.03 kg during at least 12 weeks [115]. In a randomized, controlled, phase 1b trial, Cagrilintide use reduced mean body weight significantly compared to the placebo group [116]. This trial, based in the US, followed patients over the course of 25 weeks, with 16 weeks of drug escalation, 4 weeks of finalized dose treatment, and 5 weeks of follow up [116]. In this trial, patients who received 1.2 mg and 2.4 mg of Cagrilintide had mean body weight reductions of 15.7% and 17.1%, respectively, compared to the placebo group, with reductions of 9.8% at week 20 [116]. Finally, in a phase 2 clinical trial, the combination of Cagrilintide and Semaglutide showed the most significant reductions in body weight with a mean difference of −15.6% versus Cagrilintide or Semaglutide treatment alone; these yielded −8.1% and −5.1% respectively [112].

Regarding tolerability, Cagrilintide’s adverse effects range from mild to moderate but maintain a low overall risk profile [116]. The adverse events reported surrounding Cagrilintide’s use were mainly gastrointestinal symptoms such as nausea, as well as superficial reactions to the location of the medication administration [117]. Although it is still under investigation, Cagrilintide has shown immense promise and, with more research, may prove to be the next horizon for weight loss management [117].

### 4.2. Bimagrumab

Bimagrumab is a human monoclonal antibody that binds to the ActRII signaling pathway, blocking it [118]. ActRII signaling blockage via this antibody effectively increases body muscle mass [118]. Bimagrumab is proving to be a novel therapy based on its mechanism of action, which involves impacting not only body fat, but also body muscle mass [119]. Bimagrumab is a class of drugs made via a monoclonal antibody [118]. Monoclonal antibodies are precise drugs, as they are immunoglobulins which are extremely specific for one epitope or antigen [120]. Most weight loss medications on the market target body fat, metabolism, or hormone pathways that can induce or reduce hunger; however, Bimagrumab is original in its mechanism of action by blocking the ActRII signaling as a human anti-ActRII antibody [118]. In clinical settings, Bimagrumab is anticipated to be administered via intravenous infusion, similar to other monoclonal antibody therapies. In a study performed on diet-induced obese mice, Bimagrumab, used in combination with Semaglutide, showed a reduction in fat mass while also showing a ~10% increase in lean mass [121]. In the same study, Semaglutide alone showed significant muscle and fat mass decreases, whereas using Bimagrumab preserved and increased muscle mass [121]. In a phase 2 randomized clinical trial that lasted 48 weeks, Bimigrumab showed a larger decrease in body fat mass than the group who received the placebo [119]. This trial was specifically for adults with type 2 diabetes, BMIs of 28–40, and HbA1c levels of 6.5% to 10.0% [119]. The promising data came from nine different locations in the US and the UK. Due to the protective nature of its mechanism of action, Bimagrumab is generating excitement in the clinical community. It is well tolerated and able to decrease fat mass and uniquely increase muscle mass, thus proving to be a pioneering treatment, especially for those unable to tolerate the mechanism of action of other weight loss medications [122].

## 5. Medications Withdrawn from the Market

### Lorcaserin (Belviq)

Lorcaserin is a medication that was used for weight loss until it was pulled from the market after the findings of a clinical trial which showed increased development of cancers in patients taking the medication. Lorcaserin works as a serotonergic agonist, specific to the 5HT2C receptor [123]. The 5HT2C receptor is a serotonin receptor that controls crucial functions in maintaining homeostasis, such as food intake [124]. Although initial research showed promise, Lorcaserin is no longer on the market as of 2020 [125]. In 2012, Lorcaserin was approved for patients with obesity as a medication to use while also undergoing lifestyle modifications, such as more physical activity along with a calorie-deficient diet [125]. As a part of the approval process, the FDA required the manufacturer of Lorcaserin to conduct a randomized, double-blind, placebo-controlled clinical trial to continue monitoring the drug’s safety [126]. This trial showed that 7.7% of the Lorcaserin group developed cancers as opposed to 7.1% in the placebo group [126]. Lorcaserin contributed to an overall increase in cancers, specifically lung, pancreatic, and colorectal cancers [126]. Although a 0.6% increase in cancers may not sound significant, 462 patients treated with Lorcaserin developed 520 primary malignancies compared to the placebo group, which had 423 patients who developed 470 primary cancers [126]. Lorcaserin was advised to be pulled from the market by the FDA in 2020 due to the findings of this clinical trial, and it has not been available since [126]. It is paramount to the safety of patients that we continue to monitor drugs, even after they have been brought to market.

## 6. Artificial Intelligence (AI) in Weight Loss Treatment

AI has become a novel part of everyday life, from ChatGPT to Snapchat’s AI for users. AI is revolutionizing the scientific world, especially within the medical field. AI is currently under investigation for implementation in diagnostic techniques, data gathering, and personalized medicine, which may be essential in weight loss management [127]. As each patient may require a different and precise approach, AI could be essential in precision medicine for weight loss. However, targeted approaches to weight loss management with AI would need to be examined clinically before being released to the public. Currently, the main categories of weight loss interventions are lifestyle modifications, including changing diet, physical activity, mental/emotional well-being, medication, and surgery. AI has enormous potential to help patients tailor their lifestyle changes to their needs. A scoping review on AI use in weight loss found that AI can assist in personalized weight loss when patients input the correct metrics (goals, lifestyle behaviors, etc.) [128]. However, inputting all the proper metrics brings up a downside to using AI: taking the time to input all the data is the patient’s responsibility. Most of the research performed on AI use in weight loss management has said that although AI has vast potential in this area, it has yet to be implemented in such a way as to make a significant impact [129]. In a promising study conducted in 2022, an AI robot could help the user make weight loss decisions [130]. In another study performed in 2023, the results showed that weight-management experts agreed with the modifications and predictions made by the machine learning model, which would prove an exciting leap for AI use in weight loss management programs [131].

## 7. Comparison Between Different Weight Loss Management Modalities

Table 1 provides a comprehensive comparison of different weight management modalities, including lifestyle interventions, pharmacotherapy, bariatric surgery, and endoscopic procedures. It highlights their mechanisms, efficacy, suitability, and limitations to assist clinicians and researchers in making informed decisions.

## 8. Safety, Efficacy, and Regulatory Considerations

Generally, the current anti-obesity medications (AOMs) have shown a significant effect on weight loss, as one year of AOMs can result in at least 5% weight reduction, which can improve many obesity-associated comorbidities [171]. Also, they achieved the same weight reduction percentage in 44–75% of 29,018 patients [171]. Nevertheless, the most commonly reported adverse effects of AOMs are dry mouth, nausea, diarrhea, and constipation, which are generally manageable [172,173,174,175,176]. On the other hand, some serious adverse events have been associated with these drugs, such as cardiovascular disease, acute kidney failure, and kidney injury [177,178]. There are some contraindications for the use of AOMs, mainly for pregnant or breastfeeding women and patients with severe renal or hepatic impairments. On the other hand, AOMs can be prescribed with extra caution in patients with non-severe renal or hepatic impairments and elderly patients. However, there are limited studies on the latter [19]. Since most clinical trials monitor drug-induced risks for only one year, we recommend further investigations of drug safety in the long term.

Regulatory oversight of AOMs is crucial to ensure their safety and efficacy before and after approval, and it involves multiple steps, from clinical trials to drug approval and post-market surveillance. According to the Food and Drug Administration (FDA), the first two phases of clinical trials should evaluate the pharmacokinetics of AOMs, their maximum doses at which they become active compared to placebo, and their ability to achieve weight loss ≥ 5% of the baseline weight. Then, phase 3 clinical trials examine the efficiency and safety profile of AOMs in randomized, double-blinded, and placebo-controlled trials. The effect on obesity-related morbidities should also be assessed throughout all phases [179]. The efficacy criteria for AOMs are either that the difference in average weight loss between the drug- and placebo-treated groups being ≥5% or the drug-treated group achieving a weight loss of ≥5% from baseline, with at least 35% of participants reaching this threshold, double the proportion of the placebo-treated group [179,180]. Both outcomes should follow one year of AOMs and be statistically significant [179,180]. The safety estimation should be based on 3000 participants in a randomized one-year trial, with at least 1500 subjects given a placebo [179]. Furthermore, given the critical role of post-market surveillance, we advise clinicians to report adverse drug reactions (ADRs) to their local regulatory agency and to encourage patients to inform their healthcare providers of any suspect ADRs.

Some recommendations can be followed to improve access to AOMs. First, they should be covered by health insurance as a core element rather than a non-essential extra. In addition, the overpricing of AOMs can hinder their access to patients and impose financial burdens on them and the healthcare system. Therefore, AOMs pricing should reflect their effectiveness and the uncertainty surrounding long-term benefits, with initial prices set reasonably low until further evidence validates their long-term benefits. Furthermore, since not all people are qualified for AOMs, it is crucial to establish a set of criteria that informs treatment allocation for those who are indicated. Although BMI remains the main measure for assessing obesity, experts emphasize the need for additional measures of obesity severity for routine clinical use to aid in identifying individuals at an increased risk of obesity-related complications, allowing for better prioritization of treatment and subsequently expanding access to AOMs among those who need them most [181].

Moreover, medical specialty societies are recommended to create and distribute educational materials that allow AOMs prescription to qualified patients by a wide range of clinicians rather than weight loss specialists exclusively [181]. When it comes to prescribing AOMs, the individual response to AOMs is variable, so prescribers should account for the MOA, safety, adverse effects, contraindications, drug interactions, and route of administration. Also, affordability should be considered with patients when selecting from available options and re-evaluated periodically. If AOMs fail to achieve clinically meaningful weight loss, other factors like the dose, adherence, behavioral change challenges, and mental or medical conditions should be taken into account, and the prescribed AOM should be stopped if it does not result in ≥5% weight loss within 3 months (4 months for naltrexone/bupropion combination therapy). Currently, there is no available method to predict which AOM will be the most effective for an individual patient. However, advancements in the field of personalized medicine, such as hormonal and genetic profiling, could make this possible. Thus, investment in these areas would be highly valuable [182].

## 9. Conclusions

With the rising prevalence of obesity and its associated comorbidities, there is a growing motivation to develop innovative solutions to address this issue. One of them is lifestyle modification, which is not always sufficient to resolve obesity, necessitating more potent interventions like anti-obesity medications AOMs and surgical procedures.

Developing new AOMs has been challenged by the efficacy and safety profiles that AOMs must achieve to be considered safe and effective. Over the past 20 years, many AOMs have emerged with mechanisms of actions that reduce hunger, promote satiety, and increase energy expenditure, leading to weight loss. Some of these drugs were made available in parenteral and peroral forms, and others were approved as combination drugs. However, some were withdrawn from the market, notably Lorcaserin, due to the risk of cancer. AOMs have managed to achieve a significant reduction in obesity and its associated complications, including diabetes, cardiovascular diseases, and sleep apnea. However, there are some side effects associated with these drugs, commonly reported as dry mouth, insomnia, and gastrointestinal symptoms, or severe adverse effects like pancreatitis, gallbladder disease, or liver injury. Thus, regulatory oversight is critical to ensure high standards of safety and efficacy.

Moreover, bariatric surgeries and endoscopic weight loss procedures have become viable options for patients requiring interventions beyond AOMs and lifestyle changes. The landscape of obesity management is rapidly evolving, with novel AOMs in clinical trials showing promising safety and efficacy profiles. Additionally, the emerging artificial intelligence (AI) has demonstrated potential benefits by personalizing weight loss interventions for patients and monitoring their progression. 

## Figures and Tables

**Table 1:** Comparison of weight management modalities: mechanisms, efficacy, and considerations.

Modality Type	Description	Efficacy and Timeframe	Patient Suitability	Side Effects and Adherence	Cost (Approx.)	References
Lifestyle interventions	Low-calorie diet (500–750 kcal/day), aerobic and resistance training, behavior therapy	6–11% weight loss in 1 year	BMI 25–29.9 (no comorbidities) or BMI ≥ 30 with comorbidities	Non-adherence: 21–60%, barriers include time and motivation	N/A	[77,132,133,134,135,136,137]
Dietary readjustment—ketogenic diet	High-fat, low-carbohydrate diet shifts metabolism toward fat oxidation and ketone production; ketones suppress ghrelin, reduce appetite, and promote weight loss.	14.5–30 kg weight loss over 1 year	No contraindications (e.g., pancreatitis, hepatic failure, fat metabolism or carnitine disorders, porphyrias). Use with caution in diabetics on insulin/oral hypoglycemics due to hypoglycemia risk.	Hypertriglyceridemia, transient hyperuricemia, hypercholesterolemia, renal stones, iron-deficiency anemia	N/A	[138,139,140,141,142,143,144,145,146]
Pharmacotherapy	GLP-1 agonists (Semaglutide, Liraglutide), lipase inhibitors (Orlistat), MC4 agonists, appetite suppressants, insulin sensitizers	4–18.6% weight loss in 6–12 months	BMI ≥ 30 or BMI ≥ 27 with comorbidities	Adherence: 10–40%, side effects: nausea, headache, constipation	USD 11–USD 1576/month	[77,147,148,149,150,151,152,153,154,155]
Bariatric surgery	Gastric bypass, sleeve gastrectomy, gastric banding, duodenal switch	17–28% weight loss over 1–2 years 12–25% weight loss over 3–10 years 18% weight loss sustained over 20 years	BMI ≥ 40 or BMI ≥ 35 with comorbidities	Side effects: nutritional deficiency, reflux, obstruction, bone loss	USD 7423–USD 33,541	[77,156,157,158,159,160,161,162]
Endoscopic procedures	Intragastric balloons, endoscopic sleeve gastroplasty (ESG), POSE, AspireAssist	4.95–21% weight loss in 1 year	BMI ≥ 30 or ≥27 with comorbidities	Side effects: nausea, vomiting, heartburn, pulmonary risks	USD 4105–USD 11,411/QALY	[77,163,164,165,166,167,168,169,170]
Combination/Adjunctive Strategy	Simultaneous or sequential use of two or more therapies (e.g., lifestyle + pharmacotherapy, endoscopy + behavioral intervention) to improve long-term outcomes and adherence	6–13% total weight loss, depending on combination and adherence	Individuals with insufficient response to monotherapy or those at high risk of dropout. BMI ≥ 30 with comorbidities	Adherence improves with behavioral support; side effects depend on pharmacologic or surgical components	Variable depending on treatment mix; often higher than monotherapy alone	[132,133,147,149]

## Abbreviations

The following abbreviations are used in this manuscript:

5HT2C	5-hydroxytryptamine 2C receptor
AACE	American Association of Clinical Endocrinologists
ACC	American College of Cardiology
ACE	American College of Endocrinology
ADRs	Adverse Drug Reactions
AHA	American Heart Association
AI	Artificial Intelligence
AOMs	Anti-Obesity Medications
AgRP	Agouti-Related Peptide
BBS	Bardet–Biedl Syndrome
BMI	Body Mass Index
CART	Cocaine- and Amphetamine-Regulated Transcript
CVDs	Cardiovascular Diseases
DALYs	Disability-Adjusted Life Years
DBP	Diastolic Blood Pressure
DPP-4	Dipeptidyl Peptidase-4
FDA	U.S. Food and Drug Administration
GLP-1	Glucagon-Like Peptide-1
HDL	High-Density Lipoprotein
LEPR	Leptin Receptor
MAO	Monoamine Oxidase
MC4R	Melanocortin-4 Receptor
NPY	Neuropeptide Y
PCOS	Polycystic Ovary Syndrome
PCSK1	Proprotein Convertase Subtilisin/Kexin Type 1
POMC	Proopiomelanocortin
QALY	Quality-Adjusted Life Year
RCTs	Randomized Control Trials
SBP	Systolic Blood Pressure
T2D	Type 2 Diabetes
TBW	Total Body Weight
TOS	The Obesity Society
TRG	Triglycerides
WHO	World Health Organization
